# The Effects of Long-Term High Water Iodine Levels in the External Environment on the Carotid Artery

**DOI:** 10.1007/s12011-021-02872-2

**Published:** 2021-08-31

**Authors:** Ji Bian, Man Zhang, Feng Li, Jie Gao, Zhexue Wei, Zijing Liu, Xiaoming Wang, Wen Jiang, Jianchao Bian

**Affiliations:** 1 Shandong Normal University, Jinan, 250014 China; 2 Shandong Institute of Prevention and Control for Endemic Disease, Jinan, 250014 China; 3 The First Affiliated Hospital of Shandong First Medical University (Shandong Provincial Qianfoshan Hospital), Jinan, 250014 China; 4 Maternal and Child Health Care Hospital of Shandong Province, Jinan, 250014 China

**Keywords:** Iodine excess, Carotid artery, Intima-media thickness, Atherosclerosis

## Abstract

Iodine excess typically affects thyroid function in the human body and may damage carotid artery. Four investigation plots with different water iodine levels were selected in Shandong Province, China. These included a low, medium, and high iodine group and an iodine excess group whose water iodine content was < 10, 50–150, 150–300, and > 300 μg/L, respectively. Residents aged 20–65 years answered a questionnaire and underwent carotid artery ultrasonography, and their height, weight, and urinary iodine concentrations were measured. A total of 2026 individuals participated in the study. Urinary iodine concentration increased with increased water iodine levels. The medial thickening rate and intimal roughness rate in the iodine excess group were significantly higher than in the other three groups. After controlling for factors such as gender, age, and BMI, iodine excess remained as a risk factor for carotid intima-media thickening. Excess water iodine in the external environment is a risk factor for intima-media thickening of the carotid artery, suggesting that iodine excess may cause vascular injury and promote atherosclerosis.

## Introduction

Excessive iodine intake may cause disease, and its sources typically include food, water, and iatrogenesis. In 1965, Suzuki[1] reported that seaweed intake was responsible for the high prevalence of goiter in Hokkaido, Japan. Similarly, in 1978, a study found that excess water iodine caused goiter in China[2]. Iatrogenic iodine excess most commonly occurs as a side effect of drugs, among which amiodarone is a common example[3]. Iodine excess typically affects thyroid function in the human body and may cause diseases such as goiter, hyperthyroidism, hypothyroidism, and autoimmune thyroid disease[4, 5]. Some studies have also found that iodine excess affects intellectual development[6]. However, the effects of iodine excess on the cardiovascular system are still largely undetermined.

Budd-Chiari syndrome (BCS) is a post-hepatic portal hypertension caused by hepatic vein and/or inferior vena cava obstruction. It is often accompanied by inferior vena cava hypertension. Researchers have reported that areas with a high incidence of BCS in China overlapped with areas with high water iodine levels and concluded that the incidence of BCS was associated with high water iodine levels[7]. An epidemiological investigation of an area with high water iodine levels found that the incidence and severity of carotid artery atherosclerosis in adults from that area were higher than of adults from an area without high water iodine levels[8]. Cell experiments also showed that iodine excess may damage rat aortic endothelial cells[9]. The carotid artery is the most vulnerable site for the development of atherosclerosis and a sensitive index of vascular diseases. Intima-media thickness (IMT) of the carotid artery is a marker of atherosclerosis in the coronary artery[10] and other blood vessels[11, 12], as well as a predictor of cardiovascular events[13]. In this study, the rate of the intima-media thickening of the carotid of individuals living in areas with different water iodine levels was investigated to explore the injuries to the arteries that are caused by high water iodine levels in the external environment.

## Methods

### Subjects

Four investigation plots with different water iodine contents were selected in Shandong Province: (1) low iodine group with water iodine levels of < 10 μg/L, (2) medium iodine group with water iodine levels of 50–150 μg/L, (3) high iodine group with water iodine levels of 150–300 μg/L, and (4) iodine excess group with water iodine levels of > 300 μg/L. Three to five nearby villages were randomly selected within each investigation plot, and all the adults who met the inclusion criteria were included as study participants.

Inclusion criteria are as follows: (1) adults aged 20 to 65 years old; (2) farmers, who resided in the local area for at least 10 years and had no history of relocation; (3) mainly drank the same water source, ate locally produced food and vegetables, and had no other iodine supplementation; (4) agreed to participate in the study; and (5) had no other endemic diseases such as endemic fluorosis.

### Survey Areas

Xianrenqiao, Dazhuang, Xiaoliu, and Xiaozhuang village in Dingzhuang Town and Huiwang village in Huiwang Town in Dezhou City were selected as the low iodine area. Guandaosun and Qiaojia village of Huiwang Town and Kangjia village of Shentou Town in Dezhou City were designated as the medium iodine area. Yangji, Zhengjia, Houzhang, and Wujia village in Huiwang Town and Liman village in Daliu Town in Dezhou City were designated as the high iodine area. Jiaochangli, Nanguannan, and Sanli village in Dangyi Town and Dongguqiao and Pihuli village in Liangcun Town in Liaocheng City were selected as the iodine excess area.

### Data Collection

Epidemiologic field investigation included the gathering of general information (i.e., name, gender, age, and occupation) and a physical examination (i.e., height, weight, urinary iodine, and carotid artery ultrasonography).

Urinary iodine: Urine samples were randomly taken on the spot, and the urinary iodine was determined by As-Ce catalysis spectrophotometry. Urine samples were collected and stored at − 20 °C and were tested in 1 week.

Carotid artery ultrasonography[14]: The morphology and hemodynamic parameters of the carotid artery were measured by a GE LOGIQ E ultrasound system. These included the (a) internal diameter of the carotid artery (D, mm): Internal diameter in diastole measured at a distance of 15 mm from the bifurcation of the common carotid artery; (b) intima-media thickness (IMT) of the carotid artery (mm): Posterior wall of the carotid artery in diastole at a distance of 10 mm from the carotid sinus; (c) carotid artery blood flow velocity: Peak systolic velocity (PSV), end diastolic velocity (EDV). Resistance index (RI) = (PSV-EDV)/PSV, pulsatility index (PI) = 2 × (PSV-EDV)/(PSV + EDV); and (d) intima–media thickening of the carotid artery: IMT ≥ 1.0 mm on one or both sides of the bilateral carotid artery.

### Statistical Analysis

The normality of data distribution was tested using the Kolmogorov–Smirnov test. Normally distributed data were presented as means ± standard deviations, and the non-normally distributed data were presented as medians and 25th and 75th percentiles. Between-group differences in age and carotid artery hemodynamic indexes were assessed using analysis of variance (ANOVA). Between group differences in gender, drinking, smoking, history of hypertension, history of diabetes, body mass index (BMI), intima-media thickening rate, and carotid artery roughness were assessed using the Chi-square test, and urinary iodine was analyzed using the Kruskal–Wallis method. Intima-media thickening of the carotid artery present was used as the dependent variable in logistic regression analysis. Data were sorted and analyzed using IBM SPSS Statistics for Windows, version 20 (IBM Corp., Armonk, N.Y., USA). The *p* < 0.05 was regarded as statistically significant.

## Results

A total of 2026 individuals participated in the study, of which 490, 489, 549, and 498 were in the low iodine group, medium iodine group, high iodine group, and iodine excess group, respectively. There were no between-group differences in age distribution, and the number of females was higher than that of males in total. Participants’ select characteristics are displayed in Table 1.

**Table 1:** Characteristics of the study participants, by group

Variable	Low (< 10 µg/L)	Medium (10–150 µg/L)	High (150–300 µg/L)	Excessive (> 300 µg/L)
Gender				
Male	184	153	213	175
Female	306	336	336	323
Age	49.89 ± 9.17	48.27 ± 9.70	49.64 ± 10.05	48.98 ± 9.60
Wine				
Never	346	368	379	371
Drinking	135	115	149	116
Ever	9	6	21	11
Smoking				
Never	361	401	394	396
Smoking	106	71	131	81
Ever	23	17	24	21
Prevalence of hypertension	25.51%	18.20%	21.68%	27.31%
Prevalence of diabetes	4.49%	3.89%	4.37%	6.63%
BMI				
< 24	178	186	230	154
24–28	198	173	183	203
≥ 28	114	130	136	141

BMI was calculated by height and weight and categorized as < 24 kg/m^2^, 24–28 kg/m^2^, and > 28 kg/m^2^, according to guidelines for the prevention and control of overweight and obesity in Chinese adults

### Urinary Iodine in Each Group

The distribution of urinary iodine in each group differed significantly, and the median urinary iodine concentration in each group increased with the increase in water iodine content. Participants in the low iodine group showed mild iodine deficiency. In contrast, those in the medium and high iodine groups had appropriate urinary iodine concentrations, and participants in the iodine excess group had excessive urinary iodine concentrations. Details are shown in Table 2.

**Table 2:** Distribution of urinary iodine, by group

Percentile	Low (µg/L)	Medium (µg/L)	High (µg/L)	Excessive (µg/L)
25	61.50	144.25	171.30	517.95
50	93.20	235.60	276.80	829.80
75	157.40	372.40	488.50	1264.95

### Morphology and Hemodynamic Indexes of the Carotid Artery in Each Group

#### The internal diameter of the carotid artery

The internal diameters of both sides of the carotid artery in the low iodine group were significantly higher than those in the other three groups. The internal diameters of the right carotid artery in the high iodine group and the iodine excess group were significantly higher than that of the medium iodine group.

### PSV and EDV

The PSV of the left carotid artery in the iodine excess group was significantly higher than that of the other three groups, and the EDV of the right carotid artery in the iodine excess group was significantly lower than that of the low iodine group and the high iodine group.

### RI and PI

The RI of the left carotid artery in the iodine excess group was significantly higher than that of the low iodine group and the high iodine group. The PI of the bilateral carotid arteries was significantly higher than that of the other three groups.

### IMT

The IMT of the bilateral carotid arteries in the iodine excess group were higher than those of the other three groups, the left carotid artery in the low iodine group was higher than the medium iodine group, and the right carotid artery in the low iodine group was higher than the medium iodine group and the high iodine group. The rate of intima-media thickening and roughness of the carotid in the iodine excess group were significantly higher than those of the other three groups, and the intima-media thickening rate in the high iodine group was higher than that in the low iodine group. Further details are provided in Table 3.

**Table 3:** Morphology and hemodynamic indexes of the carotid artery, by group

	Variable	Low (< 10 µg/L)	Medium (10–150 µg/L)	High (150–300 µg/L)	Excessive (> 300 µg/L)
Left	D (mm)	6.3 ± 0.74	5.86 ± 0.74^*^	5.89 ± 0.74^*^	5.99 ± 0.86^*#&^
	PSV (cm/s)	73.50 ± 15.96	73.76 ± 18.59	73.71 ± 18.31	80.61 ± 19.19^*#&^
	EDV (cm/s)	22.06 ± 6.23	21.09 ± 6.27	22.07 ± 6.92	22.05 ± 7.88
	RI	0.69 ± 0.08	0.71 ± 0.07	0.70 ± 0.08	0.72 ± 0.14^*&^
	PI	1.08 ± 0.18	1.11 ± 0.18^*^	1.08 ± 0.19	1.14 ± 0.23^*#&^
	IMT	0.67 ± 0.13	0.63 ± 0.16^*^	0.65 ± 0.20	0.74 ± 0.26^*#&^
Right	D (mm)	6.43 ± 0.71	5.85 ± 0.75^*^	5.99 ± 0.79^*#^	6.05 ± 0.74^*#^
	PSV (cm/s)	72.79 ± 16.28	70.67 ± 18.24	72.79 ± 17.99	73.29 ± 17.71
	EDV (cm/s)	21.21 ± 5.80	20.84 ± 7.14	21.21 ± 6.09	19.97 ± 7.83^*&^
	RI	0.70 ± 0.07	0.70 ± 0.08	0.70 ± 0.08	0.71 ± 0.19
	PI	1.10 ± 0.17	1.09 ± 0.19	1.10 ± 0.17	1.14 ± 0.24^*#&^
	IMT	0.67 ± 0.13	0.63 ± 0.15^*^	0.64 ± 0.16^*^	0.75 ± 0.23^*#&^
Intima-media thickening		4.24%	5.70%	7.54%^*^	22.81%^*#&^
Roughness rate		31.53%	35.63%	31.48%	42.38%^*#&^

*D* internal diameter of the carotid artery, *PSV* peak systolic velocity, *EDV* end diastolic velocity, *RI* resistance index, *PI* pulsatility index, *IMT* intima-media thickness of the carotid artery. *p < 0.05 vs. low group, ^#^*p* < 0.05 vs. medium group, ^&^*p* < 0.05 vs. high group

**Table 4:** Logistic regression analysis of carotid intima-media thickening

Index	Coefficient of partial regression β	Wald χ^2^	*p* Value	OR	95% Confidence interval	
Iodine group		121.130	0.000			
Low	Reference					
Medium	0.491	2.100	0.147	1.634	0.841	3.176
High	0.579	4.169	0.041	1.785	1.023	3.113
Excessive	2.133	72.190	0.000	8.443	5.161	13.811
BMI		7.905	0.019			
< 24 kg/m^2^	Reference					
24–28 kg/m^2^	-0.021	0.012	0.912	0.979	0.670	1.429
> 28 kg/m^2^	0.456	5.089	0.024	1.578	1.062	2.347
Age		35.525	0.000			
20–40	Reference					
40–50	0.341	1.145	0.285	1.406	0.753	2.624
50–60	0.949	9.713	0.002	2.582	1.422	4.689
60–65	1.515	21.923	0.000	4.550	2.413	8.579
Hypertension	0.783	22.177	0.000	2.189	1.580	3.032
Smoking		7.421	0.024			
No smoking	Reference					
Smoking at present	0.627	7.420	0.006	1.872	1.192	2.938
Smoking in the past	0.380	1.023	0.312	1.462	0.701	3.050
Constant	-4.370	101.820	0.000	0.013		

BMI was calculated by height and weight and categorized as < 24 kg/m^2^, 24–28 kg/m^2^, and > 28 kg/m^2^, according to guidelines for the prevention and control of overweight and obesity in Chinese adults. Age was categorized as 20–40, 40–50, 50–60, and 60–65 years old

#### Regression Analysis

Logistic regression analysis was carried out with the presence of carotid intima-media thickening as the dependent variable. After adjusting for variables such as gender, diabetes history, and drinking habits, iodine excess was a risk factor for carotid intima-media thickening. In addition, obesity, history of hypertension, smoking, and age > 50 years were all risk factors for carotid intima-media thickening Table 4.

## Discussions

Carotid artery IMT is a noninvasive measurement of arterial wall thickness. It is considered to be a marker of atherosclerosis and is associated with its final outcomes, including myocardial infarction and stroke[10, 15, 16]. Some studies have also shown that carotid artery IMT is closely associated with ischemic heart disease and stroke. In the Multi-Ethnic Study of Atherosclerosis study[14], IMT measurements were used as alternative indicators of subclinical cardiovascular diseases and predictors of cardiovascular events. Our current study found that water iodine levels > 300 μg/L were an independent risk factor for carotid intima-media thickening. When the water iodine content exceeded 300 μg/L, the intima-media thickening rate of carotid artery in the population significantly increased, suggesting that excessive iodine may cause cardiovascular system damages.

Changes in thyroid hormone levels may play an important role in vascular injuries induced by excessive iodine. Thyroid hormones can regulate body energy and lipid metabolism and affect cardiac function and blood lipids; they are also important metabolic regulators of cardiovascular activity and can act on cardiomyocytes, vascular smooth muscle, and endothelial cells[17, 18]. Long-term excessive iodine intake can lead to hypothyroidism or hyperthyroidism, of which hypothyroidism is dominant[19]. Both of these conditions have negative effects on the cardiovascular system.

Hypothyroidism can be manifested as bradycardia, decreased cardiac output, elevated diastolic blood pressure, and peripheral vasoconstriction[20, 21]. Identified cardiovascular risk factors include increased C-reactive protein, total cholesterol, low-density lipoprotein cholesterol (LDL-C), and decreased high-density lipoprotein cholesterol (HDL-C), all of which are also common in patients with hypothyroidism[22]. It has been reported that the IMT of the carotid artery in patients with hypothyroidism is higher than that in the control group and that a decrease in IMT was observed after one year of levothyroxine replacement therapy[23]. Studies have also shown that the thickness of the human thoracic aortic wall increased with the increase of serum thyroid stimulating hormone (TSH) concentration and pointed out that hypothyroidism might indicate the occurrence of aortic atherosclerosis[24]. Hyperthyroidism may manifest as elevated systolic blood pressure, venous resistance, increased cardiac output and myocardial weight, tachycardia, and atrial fibrillation[20, 21]. Studies have found that for adults in the iodine excess area, the free thyroxine and systolic blood pressure are higher in areas with iodine excess than that in areas with suitable iodine excess[25]. It has been reported that IMT of the carotid artery increases with thyroid hormone levels and that hyperthyroidism is an independent risk factor for IMT increase by multivariate regression[26].

In addition to regulating hormone levels, excess iodine can also directly promote vascular injury. Some studies have shown that excessive iodine intake can lead to an increase in blood glucose and blood pressure, as well as a decrease in HDL-C levels and an increase in LDL-C levels[27], thus affecting the cardiovascular system. Our current study also found that the prevalence of diabetes in the iodine excess group was higher than that of the other three groups and that the prevalence of hypertension in the iodine excess group was higher than that in the medium iodine group and the high iodine group. These findings are consistent with previous studies. Animal experiments have found that iodine excess could increase lipid peroxidation levels in the thyroid, liver, and blood of mice, suggesting that excessive iodine may damage blood vessels through oxidation[28]. In addition, cell experiments have shown that excessive iodine exposure could increase oxidative stress and alter the expression of adhesion factors and nitric oxide synthase activity, resulting in vascular endothelial cell injury[9].

The PI reflects cerebral vascular compliance and elasticity, while the RI reflects cerebral vascular resistance; these two indexes can be used as sensitive indicators for the diagnosis and prognosis of ischemic cerebrovascular disease[29]. This study found that the carotid artery RI and PI increased in the iodine excess group, indicating that the elasticity and compliance of the carotid artery decreased. In addition, intimal roughness increased in the iodine excess group, suggesting that the intima was damaged. The above morphological indexes and hemodynamic changes of the carotid artery in the iodine excess group suggested that the risk of further developing atherosclerosis was higher than that of the other groups.

At present, few studies have investigated the effects of iodine excess on the cardiovascular system, especially on the carotid artery. This current study explored the damage to the carotid artery caused by high water iodine in the external environment and generated ideas for the study of the effects of iodine excess on atherosclerosis. In addition to iodine excess, this study also found that obesity, hypertension, age > 50 years old, and smoking are also risk factors for intra-media thickening. This finding is consistent with the results of previous studies and enhances the reliability of this study.

This population-based study found that iodine excess may affect carotid artery morphology and hemodynamic indexes. The possible mechanisms that underlie these associations include the function of thyroid hormones and direct injury to blood vessels by iodine; however, the specific mechanism is still unclear. In addition, the water iodine groupings in this study were relatively wide, and the precise cutoff for the water iodine concentration that causes damage to the carotid artery needs to be further studied.

## Conclusion

This study found that excessive iodine in the external environment is a risk factor for carotid intima-media thickening, suggesting that iodine excess may promote atherosclerosis. Its effects on the cardiovascular system should be taken into account when discussing the effects of iodine excess on human health. Future studies should explore the role of thyroid hormones and the existence of other pathways through which iodine excess may affect the cardiovascular system.

## Data Availability

All data generated or analyzed during this study are included in this published article.
